# Immunogenicity and reactogenicity of ten-valent versus 13-valent pneumococcal conjugate vaccines among infants in Ho Chi Minh City, Vietnam: a randomised controlled trial

**DOI:** 10.1016/S1473-3099(18)30734-5

**Published:** 2019-05

**Authors:** Beth Temple, Nguyen Trong Toan, Vo Thi Trang Dai, Kathryn Bright, Paul Vincent Licciardi, Rachel Ann Marimla, Cattram Duong Nguyen, Doan Y Uyen, Anne Balloch, Tran Ngoc Huu, Edward Kim Mulholland

**Affiliations:** a Division of Global and Tropical Health, Menzies School of Health Research, Darwin, NT, Australia; b Department of Infectious Disease Epidemiology, London School of Hygiene & Tropical Medicine, London, UK; c Department of Infection and Immunity, Murdoch Children's Research Institute, Melbourne, VIC, Australia; d Department of Disease Control and Prevention, Pasteur Institute of Ho Chi Minh City, Ho Chi Minh City, Vietnam; e Department of Microbiology and Immunology, Pasteur Institute of Ho Chi Minh City, Ho Chi Minh City, Vietnam; f Department of Paediatrics, University of Melbourne, Melbourne, VIC, Australia

## Abstract

**Background:**

Few data are available to support the choice between the two currently available pneumococcal conjugate vaccines (PCVs), ten-valent PCV (PCV10) and 13-valent PCV (PCV13). Here we report a head-to-head comparison of the immunogenicity and reactogenicity of PCV10 and PCV13.

**Methods:**

In this parallel, open-label, randomised controlled trial, healthy infants from two districts in Ho Chi Minh City, Vietnam, were randomly allocated (in a 3:3:5:4:5:4 ratio), with use of a computer-generated list, to one of six infant PCV schedules: PCV10 in a 3 + 1 (group A), 3 + 0 (group B), 2 + 1 (group C), or two-dose schedule (group D); PCV13 in a 2 + 1 schedule (group E); or no infant PCV (control; group F). Blood samples were collected from infants between 2 months and 18 months of age at various timepoints before and after PCV doses and analysed (in a blinded manner) by ELISA and opsonophagocytic assay. The trial had two independent aims: to compare vaccination responses between PCV10 and PCV13, and to evaluate different schedules of PCV10. In this Article, we present results pertaining to the first aim. The primary outcome was the proportion of infants with an IgG concentration of at least 0·35 μg/mL for the ten serotypes common to the two vaccines at age 5 months, 4 weeks after the two-dose primary vaccination series (group C *vs* group E, per protocol population). An overall difference among the schedules was defined as at least seven of ten serotypes differing in the same direction at the 10% level. We also assessed whether the two-dose primary series of PCV13 (group E) was non-inferior at the 10% level to a three-dose primary series of PCV10 (groups A and B). This trial is registered with ClinicalTrials.gov, number NCT01953510.

**Findings:**

Of 1424 infants screened between Sept 30, 2013, and Jan 9, 2015, 1201 were allocated to the six groups: 152 (13%) to group A, 149 (12%) to group B, 250 (21%) to group C, 202 (17%) to group D, 251 (21%) to group E, and 197 (16%) to group F. 237 (95%) participants in group C (PCV10) and 232 (92%) in group E (PCV13) completed the primary vaccination series and had blood draws within the specified window at age 5 months, at which time the proportion of infants with IgG concentrations of at least 0·35 μg/mL did not differ between groups at the 10% level for any serotype (PCV10–PCV13 risk difference −2·1% [95% CI −4·8 to −0·1] for serotype 1; −1·3% [–3·7 to 0·6] for serotype 4; −3·4% [–6·8 to −0·4] for serotype 5; 15·6 [7·2 to 23·7] for serotype 6B; −1·3% [–3·7 to 0·6] for serotype 7F; −1·6% [–5·1 to 1·7] for serotype 9V; 0·0% [–2·7 to 2·9] for serotype 14; −2·1% [–5·3 to 0·9] for serotype 18C; 0·0% [–2·2 to 2·3] for serotype 19F; and −11·6% [–18·2 to −4·9] for serotype 23F). At the same timepoint, two doses of PCV13 were non-inferior to three doses of PCV10 for nine of the ten shared serotypes (excluding 6B). Reactogenicity and serious adverse events were monitored according to good clinical practice guidelines, and the profiles were similar in the two groups.

**Interpretation:**

PCV10 and PCV13 are similarly highly immunogenic when used in 2 + 1 schedule. The choice of vaccine might be influenced by factors such as the comparative magnitude of the antibody responses, price, and the relative importance of different serotypes in different settings.

**Funding:**

National Health and Medical Research Council of Australia, and Bill & Melinda Gates Foundation.

## Introduction

*Streptococcus pneumoniae* (pneumococcus) is a leading vaccine-preventable cause of serious infection in young children, and was estimated to cause 294 000 deaths among children younger than 5 years of age in 2015.^1^ The greatest burden of pneumococcal disease and related mortality is in low-income and middle-income countries (LMICs).

Two pneumococcal conjugate vaccines (PCVs) are currently licensed for infant vaccination against pneumococcus. 13-valent PCV (PCV13) contains pneumococcal serotypes 1, 3, 4, 5, 6A, 6B, 7F, 9V, 14, 18C, 19A, 19F, and 23F. Ten-valent PCV (PCV10) contains ten of these serotypes (except serotypes 3, 6A, and 19A), although there is evidence for some cross-protection against serotype 6A and 19A disease.^2^, ^3^, ^4^ PCV10 and PCV13 have been shown to be immunologically non-inferior to the first-licensed, seven-valent PCV (PCV7),^5^, ^6^, ^7^ but there are few data directly comparing PCV10 with PCV13, despite these vaccines having been available for several years. A trial from Papua New Guinea compared three doses of PCV10 and PCV13 administered at 1 month, 2 months, and 3 months of age, with immunogenicity data obtained prevaccination, after dose three, and at 9 months of age.^8^ Two European trials of investigational next-generation pneumococcal vaccines have included control groups of both PCV10 and PCV13, administered in a 3 + 1 schedule at 2 months, 3 months, 4 months, and 12–15 months of age, with immunogenicity data obtained post-primary series, pre-booster, and post-booster.^9^, ^10^ Two other trials with post-primary series immunogenicity data available are registered on ClinicalTrials.gov: a trial from The Gambia of investigational, protein-based pneumococcal vaccines administered in a 3 + 0 schedule that includes both PCV10 and PCV13 control groups (NCT01262872); and a trial from Mexico to evaluate mixed regimens that includes groups that received a two-dose primary series of either PCV10 or PCV13 (NCT01641133). In addition, a small, non-randomised study from the Netherlands compared booster responses to PCV10 and PCV13 given in a 3 + 1 schedule.^11^ Broadly, these studies have shown that both PCV10 and PCV13 are highly immunogenic post-primary series and post-booster. Serotype-specific geometric mean concentrations (GMCs) of IgG antibody after vaccination with PCV13 tend to be higher post-primary series, lower pre-booster, and higher post-booster than GMCs after PCV10 vaccination, although these trends do not hold for all serotypes. Notably, of these studies, only the Papua New Guinean study^8^ and the Dutch study^11^ of the booster response were designed specifically to evaluate differences in the immunogenicity of the two vaccines.

**Research in context**

**Evidence before this study**

The licensure of the two currently available pneumococcal conjugate vaccines (PCVs), the ten-valent PCV (PCV10) and the 13-valent PCV (PCV13), was based on demonstration of their immunological non-inferiority to seven-valent PCV. However, in itself, this non-inferiority does not preclude differences between these two second-generation PCVs. We searched PubMed from inception to Feb 28, 2019, using search terms including, but not limited to, “10-valent pneumococcal conjugate vaccine” OR “13-valent pneumococcal conjugate vaccine” AND “immunogenicity”. Two studies have been published on the comparative immunogenicity of PCV10 and PCV13: one from the Netherlands comparing the booster response in a 3 + 1 schedule, and a trial of a novel schedule at 1 months, 2 month, and 3 months in Papua New Guinea. A further two European trials of investigational vaccines contained control groups that received PCV10 or PCV13 in a 3 + 1 schedule. These studies indicated that both vaccines are highly immunogenic. The vaccines differed little in terms of the proportions of children achieving protective levels of antibody, but differences in the geometric mean concentration of antibody were commonly observed and tended to favour PCV13, albeit with variations across the studies. Given the paucity of comparative data on PCV10 and PCV13, countries considering PCV introduction have little on which to base their decision, other than the relative cost of the vaccines.

**Added value of this study**

This is the first published study to compare the two currently licensed PCVs in a 2 + 1 schedule—a schedule increasingly used by low-income and middle-income countries (LMICs), and one of the WHO-recommended schedules. The results of this study will therefore have importance in LMIC settings, which often have a high burden of pneumococcal disease.

**Implications of all the available evidence**

The data from this randomised controlled trial in a LMIC support previous non-comparative data that both PCV10 and PCV13 are highly immunogenic in a 2 + 1 schedule, with similar reactogenicity. There are few differences between the two vaccines in relation to the 0·35 μg/mL correlate of protection, but the geometric mean concentrations of antibody, both post-primary series and post-booster, tend to be higher after vaccination with PCV13. It is hard to assess whether these differences would translate to differing degrees of protection afforded by the two vaccines, particularly for mucosal disease, in which a higher concentration of antibody might be required for protection. Vietnam and other LMICs considering vaccine introduction might wish to consider the immunological differences shown in this study in the context of their own pneumococcal epidemiology.

Given the few comparative data, particularly data from LMICs, available to influence the choice of PCV, we did a randomised controlled trial in Vietnam (the Vietnam Pneumococcal Project) of different infant pneumococcal vaccination schedules, including a head-to-head comparison of PCV10 and PCV13 delivered in a 2 + 1 schedule, one of the schedules recommended by WHO.^12^ The trial had two independent aims: to compare vaccination responses between PCV10 and PCV13, and to evaluate different schedules of PCV10. In this Article we present results pertaining to the first aim.

## Methods

### Study design and participants

We designed a parallel, open-label, randomised controlled trial to investigate simplified childhood vaccination schedules that are appropriate for use in LMICs. The trial was conducted in two districts within Ho Chi Minh City, Vietnam. Infants with no significant maternal or perinatal history and who were born at or after 36 weeks' gestation were enrolled at 2 months of age and followed up to 24 months of age. Infants were excluded if they had any known allergy to any component of the vaccine or had had an allergic or anaphylactic reaction to any previous vaccine, had a known immunodeficiency disorder, or were born to a mother infected with HIV. Full details of the participant eligibility criteria and recruitment processes have been described previously.^13^

A parent or legal guardian of each participant provided written informed consent. The protocol was approved by the Institutional Review Board at the Pasteur Institute of Ho Chi Minh City, Vietnam, and ethical approval was obtained from the Human Research Ethics Committee of the Northern Territory Department of Health and Menzies School of Health Research, Australia, and the Ministry of Health Ethics Committee, Vietnam. The trial was overseen by an independent data safety and monitoring board. The protocol for this trial has been published elsewhere.^13^

### Randomisation and masking

A computer-generated list of randomisation numbers was used in a block randomisation scheme, stratified by district, to allocate participants (in a 3:3:5:4:5:4 ratio) to one of six groups. This was a single-blind trial with all laboratory-based outcome assessors masked to the group allocation. Additional details of the randomisation and masking have been described previously.^13^

### Procedures

**Figure 1:**
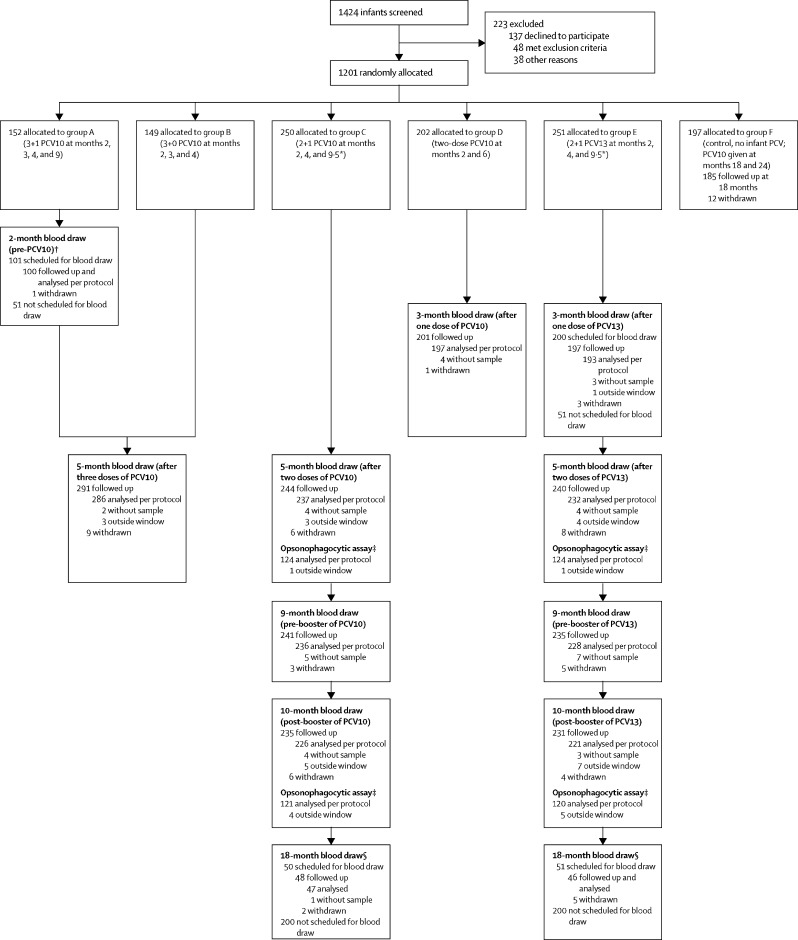
Trial profile Samples collected outside the visit window (27–43 days post-vaccination) were included only in the intention-to-treat analyses. The most common reason for participants to be without a blood sample was that the nurse was unable to successfully find a vein (18 [49%] of 37 missing blood draws). PCV=pneumococcal conjugate vaccine. PCV10=ten-valent PCV. PCV13=13-valent PCV. *PCV (and the hexavalent diphtheria, tetanus, pertussis, polio, *Haemophilus influenzae* type b, and hepatitis B [DTaP-IPV-Hib-HepB] vaccine) were administered at 9·5 months in participants from groups C and E because the Vietnamese Ministry of Health does not permit co-administration of measles and DTaP-IPV-Hib-HepB (see appendix for full schedules of PCV and co-administered vaccines). †The 2-month blood sample from group A provided pre-PCV data; samples at this timepoint were only collected from one study group, with the assumption that all groups would be interchangeable at baseline as a result of randomisation. ‡125 participants from groups C and E contributed to the opsonophagocytic assay analyses, selected as the first 125 with both post-primary series and post-booster blood samples collected. §Participants allocated to groups A–E from the last 300 recruited provided a blood sample at 18 months of age, with the remainder providing a sample at an alternative timepoint (appendix).

Participants were assigned to receive one of six infant vaccination schedules: PCV10 in a 3 + 1 (group A), 3 + 0 (group B), 2 + 1 (group C), or two-dose (group D) schedule; PCV13 in a 2 + 1 schedule (group E); or a control group (group F) that received no infant doses of PCV (figure 1). The control group was included to contribute data primarily for the secondary nasopharyngeal carriage outcomes, which will be presented elsewhere. Participants also received four doses of the hexavalent diphtheria, tetanus, pertussis, polio, *Haemophilus influenzae* type b, and hepatitis B (DTaP-IPV-Hib-HepB) vaccine. Participants in groups A–E provided four blood samples over the course of the trial. The timepoints for the collection of blood samples varied both between and within study groups to enable more questions to be addressed within the confines of a maximum of four blood samples per participant (see appendix for the full schedule of vaccines and samples). As such, the number of blood samples varied by timepoint, and samples from different PCV10 study groups contributed to analyses of the comparative immunogenicity of PCV10 and PCV13 at different timepoints: pre-PCV from group A; 4 weeks after one dose of PCV from group D; post-primary series (4 weeks after two doses of PCV), pre-booster (at 9 months of age), and post-booster (4 weeks after a booster dose of PCV at 9·5 months of age) from group C; and 18 months of age from a subset of group C (figure 1). We assessed the concentrations of serotype-specific IgG antibodies to all 13 serotypes in PCV13 using a modified third-generation standardised ELISA.^14^ Functional antibody response to all 13 serotypes were also assessed by opsonophagocytic assay.^15^

### Outcomes

To compare vaccination responses between PCV10 and PCV13, we planned to fully evaluate the immunogenicity of a 2 + 1 schedule (PCV10 or PCV13 given at 2 months, 4 months, and 9·5 months of age) in a head-to-head manner. The primary outcome was the proportion of children with protective levels of antibody (defined as ≥0·35 μg/mL, assessed by ELISA). GMCs of antibodies were also recorded. The primary outcome timepoint was 4 weeks post-primary series (age 5 months). At this timepoint, we also compared the two-dose primary series of PCV13 (group E) with a three-dose primary series of PCV10 given at 2 months, 3 months, and 4 months of age (groups A and B). This comparison was listed in the protocol as the primary outcome because, at the time the trial was designed, the two-dose primary series was not an approved schedule for PCV10. Both comparisons are presented here.

Secondary outcomes also included functional antibody responses to all 13 serotypes, assessed by opsonophagocytic assay. The proportion of children with an opsonisation index of at least 8 and geometric mean opsonisation indices were recorded in a subset of participants at 4 weeks post-primary series and 4 weeks post-booster.

The comparative reactogenicity of PCV10 and PCV13 was also evaluated. Reactogenicity assessments included erythema at the PCV and DTaP-IPV-Hib-HepB vaccination sites and axillary temperature on days 0–4 post-vaccination, as measured by the parent or caregiver and recorded on a parent-held diary card.

A post-hoc analysis comparing the proportion of children with antibody concentrations of at least 1·00 μg/mL was done post-primary series and post-booster to explore whether the use of a higher threshold of protection would identify more differences between the vaccines.

### Statistical analysis

The groups were primarily compared in terms of the proportions of children with a serotype-specific IgG concentration of at least 0·35 μg/mL at 4 weeks post-primary series (the threshold used for comparing PCV formulations). For the head-to-head comparison of two-dose primary series of PCV10 and PCV13, a 10% risk difference was considered clinically significant. Risk differences (PCV10 – PCV13) with 95% CIs were calculated with the Newcombe-Score method. The null hypothesis for each of the ten shared serotypes was that the risk difference was between −10% and 10%, with the null hypothesis rejected if the 95% CI of the risk difference was entirely outside of this range. An overall difference was considered demonstrated if at least seven of the ten individual null hypotheses were rejected in the same direction.

The two-dose primary series of PCV13 and the three-dose primary series of PCV10 were compared in terms of non-inferiority, based on a non-inferiority margin of a 10% risk difference, as used by regulatory authorities. The null hypothesis for each of the shared serotypes was that the risk difference was greater than 10%, with the null hypothesis rejected if the upper bound of the 90% CI was less than 10% (equivalent to using a 5% one-sided test). An overall conclusion of non-inferiority was drawn if the null hypotheses were rejected for at least seven of the ten shared serotypes.

The sample size provided 98% power for an overall conclusion on the difference between two doses of PCV10 and PCV13, and more than 99% power for an overall conclusion on the non-inferiority of two doses of PCV13 compared with three doses of PCV10. Details of the sample size calculations have been described previously.^13^

IgG concentrations between groups were also compared in terms of GMC ratios (PCV10 / PCV13) with 95% CIs, and were described as higher in one group if the 95% CI excluded a ratio of 1·00. Similarly, geometric mean opsonisation indexes were described as higher in one group if the 95% CI of the ratio of geometric mean opsonisation indexes (PCV10 / PCV13) excluded a ratio of 1·00. Risk differences were calculated for the proportion of children with an opsonisation index of at least 8, with a 10% difference considered significant, in line with the IgG comparisons. Beyond the primary outcome, our aim was to provide an overall description of the pattern of differences in immunogenicity between PCV10 and PCV13. As such, no formal adjustments for multiple comparisons were made, but we have deliberately avoided reporting p values. Comparisons of reactogenicity (proportions of participants with erythema or fever) between groups were done with Fisher's exact tests.

Statistical analyses were done in accordance with the protocol and the statistical analysis plan. All immunological analyses were done on the per-protocol population, and primary analyses were repeated on the intention-to-treat population. Reactogenicity analyses were done on the intention-to-treat population. Analyses were done using Stata statistical software (release 14).

The trial is registered at ClinicalTrials.gov, number NCT01953510.

### Role of the funding source

The funders of the study had no role in study design, data collection, data analysis, data interpretation, or writing of the report. The corresponding author had full access to all the data in the study and had final responsibility for the decision to submit for publication.

## Results

1424 infants were screened between Sept 30, 2013, and Jan 9, 2015, with 1201 (84%) enrolled (figure 1): 152 (13%) to group A, 149 (12%) to group B, 250 (21%) to group C, 202 (17%) to group D, 251 (21%) to group E, and 197 (16%) to group F. The groups were balanced with respect to baseline characteristics (table 1). Overall, 1179 (98%) participants completed their primary series vaccinations, 1146 (95%) received their booster dose of PCV or were followed up to 9 months of age, and 1093 (91%) were followed up to 18 months of age. Of the 108 participants withdrawn before 18 months, the reasons for withdrawal were: moved away and lost to follow-up (55 [51%]); refused a study procedure (23 [21%]); voluntary withdrawal (22 [20%]); and other (eight [8%]).

**Table 1:** Baseline characteristics by study group

		**Group A (n=152)**	**Group B (n=149)**	**Group C (n=250)**	**Group D (n=202)**	**Group E (n=251)**	**Group F (n=197)**
Sex							
	Male	66 (43%)	73 (49%)	135 (54%)	91 (45%)	127 (51%)	100 (51%)
	Female	86 (57%)	76 (51%)	115 (46%)	111 (55%)	124 (49%)	97 (49%)
District							
	4	68 (45%)	67 (45%)	112 (45%)	90 (45%)	111 (44%)	87 (44%)
	7	84 (55%)	82 (55%)	138 (55%)	112 (55%)	140 (56%)	110 (56%)
Birthweight, g*		3234 (424)	3212 (349)	3228 (370)	3234 (410)	3199 (357)	3208 (395)
Place of delivery							
	Hospital	149 (98%)	149 (100%)	245 (98%)†	194 (96%)	247 (99%)†	192 (97%)
	Other	3 (2%)	0	4 (2%)	8 (4%)	3 (1%)	5 (3%)
Type of delivery							
	Normal	89 (59%)	85 (57%)	160 (64%)	130 (64%)	151 (60%)	121 (61%)
	Elective caesarean	30 (20%)	30 (20%)	43 (17%)	36 (18%)	57 (23%)	34 (17%)
	Emergency caesarean	27 (18%)	30 (20%)	40 (16%)	34 (17%)	42 (17%)	41 (21%)
	Other or unknown	6 (4%)	4 (3%)	7 (3%)	2 (1%)	1 (0·4%)	1 (1%)
Cigarette smoker at residence							
	No	57 (38%)	52 (35%)	81 (33%)†	74 (37%)	86 (34%)	72 (37%)
	Yes	95 (63%)	97 (65%)	168 (67%)	128 (63%)	165 (66%)	125 (63%)
Breastfeeding at enrolment							
	No	41 (27%)†	42 (28%)	55 (22%)	37 (18%)	56 (22%)†	56 (29%)†
	Yes	110 (73%)	107 (72%)	195 (78%)	165 (82%)	194 (78%)	140 (71%)

Data are n (%) or mean (SD).

* Birthweight data missing for ten participants (one from group B, three from group C, three from group D, two from group E, and one from group F).

† Data missing for one participant.

**Figure 2:**
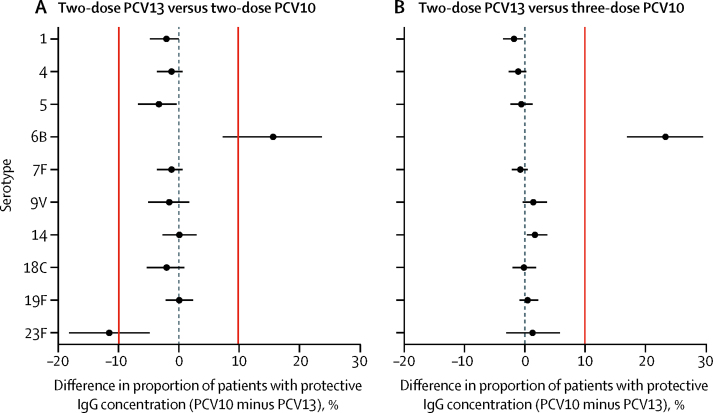
Comparative immunogenicity of PCV13 versus PCV10 at 4 weeks post-primary series Data are differences (PCV10 minus PCV13) in the proportions of patients with protective serotype-specific IgG concentrations (≥0·35 μg/mL) in patients who received PCV13 versus those who received PCV10. (A) Two-dose primary series of PCV13 (at 2 months and 4 months; group E) versus two-dose primary series of PCV10 (at 2 months and 4 months; group C). (B) Two-dose primary series of PCV13 (group E) versus three-dose primary series of PCV10 (at 2 months, 3 months, and 4 months; groups A and B). Bars represent 95% CIs for two-sided tests of difference (A) or 90% CIs for one-sided tests of non-inferiority (B), with solid vertical lines indicating the predefined thresholds for determining differences or non-inferiority between groups. PCV10=ten-valent pneumococcal conjugate vaccine. PCV13=13-valent pneumococcal conjugate vaccine.

At 5 months of age, among the 237 (95%) participants in group C (PCV10) and 232 (92%) in group E (PCV13) who completed the primary vaccination series and had blood draws within the specified time window (figure 1), the head-to-head comparison of two doses PCV13 and two doses of PCV10 showed no evidence of a difference in the proportion of infants with a serotype-specific IgG concentration of at least 0·35 μg/mL, with the CIs for the between-group differences overlapping with the −10% to 10% range for all ten shared serotypes (table 2, figure 2). In both groups, more than 95% of participants had protective IgG concentrations for all serotypes except 6B and 23F. Comparing the magnitude of the response on the basis of GMC ratio, GMCs were higher in the PCV10 group than in the PCV13 group for serotypes 6B and 19F, and higher in the PCV13 group than in the PCV10 group for the other eight shared serotypes (table 2).

**Table 2:** Post-primary series immunogenicity in the per-protocol population

	**Participants with IgG concentration ≥0·35 μg/mL, % (95% CI)**			**Risk difference, %**		**GMC, μg/mL (95% CI)**			**GMC ratio (95% CI)**	
	Two-dose PCV10 (n=237)	Three-dose PCV10 (n=286)	Two-dose PCV13 (n=232)	Two-dose PCV10 minus PCV13 (95% CI)	Three-dose PCV10 minus PCV13 (90% CI)	Two-dose PCV10 (n=237)	Three-dose PCV10 (n=286)	Two-dose PCV13 (n=232)	Two-dose PCV10 / PCV13	Three-dose PCV10 / PCV13
**Shared PCV serotypes**										
1	97·9 (95·1 to 99·3)	98·3 (96·0 to 99·4)	100·0 (98·4 to 100·0)	−2·1 (−4·8 to −0·1)	−1·7 (−3·5 to −0·3)	2·21 (1·97 to 2·48)	2·79 (2·51 to 3·10)	4·88 (4·40 to 5·42)	0·45 (0·39 to 0·53)	0·57 (0·49 to 0·66)
4	98·7 (96·3 to 99·7)	99·0 (97·0 to 99·8)	100·0 (98·4 to 100·0)	−1·3 (−3·7 to 0·6)	−1·0 (−2·6 to 0·3)	3·21 (2·87 to 3·58)	3·85 (3·44 to 4·31)	4·82 (4·41 to 5·26)	0·67 (0·58 to 0·77)	0·80 (0·69 to 0·93)
5	95·8 (92·4 to 98·0)	98·6 (96·5 to 99·6)	99·1 (96·9 to 99·9)	−3·4 (−6·8 to −0·4)	−0·5 (−2·3 to 1·3)	1·17 (1·07 to 1·27)	1·81 (1·67 to 1·97)	2·20 (2·00 to 2·41)	0·53 (0·47 to 0·60)	0·83 (0·73 to 0·94)
6B	76·8 (70·9 to 82·0)	84·6 (79·9 to 88·6)	61·2 (54·6 to 67·5)	15·6 (7·2 to 23·7)	23·4 (17·0 to 29·6)	0·80 (0·69 to 0·92)	1·08 (0·95 to 1·23)	0·48 (0·42 to 0·55)	1·65 (1·36 to 1·99)	2·24 (1·86 to 2·69)
7F	98·7 (96·3 to 99·7)	99·3 (97·5 to 99·9)	100·0 (98·4 to 100·0)	−1·3 (−3·7 to 0·6)	−0·7 (−2·1 to 0·5)	2·07 (1·89 to 2·27)	3·04 (2·79 to 3·32)	3·33 (3·05 to 3·63)	0·62 (0·55 to 0·71)	0·91 (0·81 to 1·03)
9V	96·2 (92·9 to 98·2)	99·3 (97·5 to 99·9)	97·8 (95·0 to 99·3)	−1·6 (−5·1 to 1·7)	1·5 (−0·3 to 3·7)	1·63 (1·47 to 1·81)	2·47 (2·26 to 2·71)	3·27 (2·93 to 3·65)	0·50 (0·43 to 0·58)	0·76 (0·66 to 0·87)
14	98·3 (95·7 to 99·5)	100·0 (98·7 to 100·0)	98·3 (95·6 to 99·5)	0·0 (−2·7 to 2·9)	1·7 (0·4 to 3·8)	5·86 (5·11 to 6·73)	9·76 (8·79 to 10·83)	7·99 (6·82 to 9·37)	0·73 (0·60 to 0·90)	1·22 (1·02 to 1·47)
18C	96·6 (93·5 to 98·5)	98·6 (96·5 to 99·6)	98·7 (96·3 to 99·7)	−2·1 (−5·3 to 0·9)	−0·1 (−2·0 to 1·9)	1·86 (1·64 to 2·11)	3·87 (3·47 to 4·30)	3·14 (2·84 to 3·48)	0·59 (0·50 to 0·70)	1·23 (1·06 to 1·43)
19F	99·2 (97·0 to 99·9)	99·7 (98·1 to 100·0)	99·1 (96·9 to 99·9)	0·0 (−2·2 to 2·3)	0·5 (−0·8 to 2·2)	9·54 (8·37 to 10·87)	8·34 (7·52 to 9·24)	7·67 (6·78 to 8·68)	1·24 (1·04 to 1·49)	1·09 (0·93 to 1·27)
23F	77·6 (71·8 to 82·8)	90·6 (86·6 to 93·7)	89·2 (84·5 to 92·9)	−11·6 (−18·2 to −4·9)	1·3 (−3·0 to 5·9)	0·89 (0·78 to 1·02)	1·32 (1·18 to 1·48)	1·14 (1·01 to 1·29)	0·78 (0·65 to 0·94)	1·16 (0·98 to 1·37)
**Additional PCV13 serotypes**										
3	5·9 (3·3 to 9·7)	7·0 (4·3 to 10·6)	97·8 (95·0 to 99·3)	−91·9 (−94·6 to −87·3)	−90·9 (−93·2 to −87·2)	0·10 (0·09 to 0·11)	0·11 (0·10 to 0·12)	1·53 (1·40 to 1·68)	0·07 (0·06 to 0·08)	0·07 (0·06 to 0·08)
6A	40·5 (34·2 to 47·1)	50·3 (44·4 to 56·3)	94·8 (91·1 to 97·3)	−54·3 (−60·8 to −47·0)	−44·5 (−49·7 to −38·8)	0·31 (0·28 to 0·35)	0·37 (0·34 to 0·41)	1·94 (1·69 to 2·21)	0·16 (0·14 to 0·19)	0·19 (0·16 to 0·22)
19A	70·5 (64·2 to 76·2)	68·2 (62·4 to 73·5)	98·3 (95·6 to 99·5)	−27·8 (−34·0 to −21·8)	−30·1 (−34·9 to–25·3)	0·55 (0·49 to 0·62)	0·56 (0·51 to 0·62)	3·82 (3·34 to 4·36)	0·14 (0·12 to 0·17)	0·15 (0·12 to 0·17)

Immunogenicity data at 4 weeks after two doses of PCV10 (at 2 months and 4 months of age, group C), two doses of PCV13 (at 2 months and 4 months of age, group E), or three doses of PCV10 (at 2 months, 3 months, and 4 months of age, groups A and B). GMC=geometric mean concentration. PCV10=ten-valent pneumococcal conjugate vaccine. PCV13=13-valent pneumococcal conjugate vaccine.

We also showed that two doses of PCV13 were non-inferior to three doses of PCV10 in terms of the proportion of infants with protective serotype-specific IgG concentrations, with the upper bound of the CI for the between-group difference less than 10% for nine of the ten shared serotypes (table 2, figure 2). The point estimates for the risk differences for these nine serotypes were all within −2% and 2%. The exception was serotype 6B, for which the proportion of participants achieving a protective IgG concentration was 84·6% (95% CI 79·9–88·6) in the PCV10 group compared with 61·2% (54·6–67·5) in the PCV13 group (risk difference 23·4% [90% CI 17·0–29·6]). IgG GMCs were higher in the PCV10 group than in the PCV13 group for serotypes 6B, 14, and 18C, and higher in the PCV13 group than in the PCV10 group for serotypes 1, 4, 5, and 9V. The conclusions based on the results of the per-protocol analysis and the intention-to-treat analysis did not differ (appendix).

**Figure 3:**
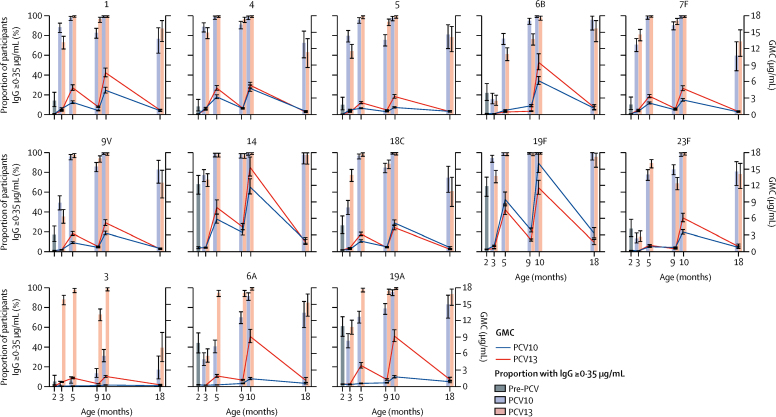
Serotype-specific IgG concentrations before and after PCV10 or PCV13 vaccinations GMCs of serotype-specific IgG (lines) and proportion of participants with protective concentrations (≥0·35 μg/mL) of serotype-specific IgG (bars) over time, for the ten shared serotypes and the three additional serotypes in PCV13, with 95% CIs. Sources of data were as follows: group A at 2 months of age (pre-PCV); group D (PCV10) and group E (PCV13) at 3 months of age (after one dose); and group C (PCV10) and group E (PCV13) at 5 months (after two-dose primary series), 9 months (pre-booster), 10 months (post-booster), and 18 months of age (in a subset of participants). GMC=geometric mean concentration. PCV10=ten-valent pneumococcal conjugate vaccine. PCV13=13-valent pneumococcal conjugate vaccine.

In addition to the post-primary series timepoint, we directly compared responses to PCV10 and PCV13 at 4 weeks after a single dose, pre-booster, 4 weeks post-booster, and 18 months of age (figure 3, appendix). At 2 months of age, pre-PCV, the highest GMCs of antibody were seen for serotypes 14 (0·64 μg/mL [95% CI 0·49 to 0·84]), 19F (0·45 μg/mL [0·39 to 0·53]), 19A (0·41 μg/mL [0·36 to 0·47]), and 6A (0·32 μg/mL [0·28 to 0·37]), and the proportion of participants with IgG concentrations of at least 0·35 μg/mL for these four serotypes ranged from 44·0% (95% CI 34·1 to 54·3), for serotype 6A, to 68·0% (57·9 to 77·0), for serotype 14 (appendix). Comparing pre-PCV and post-PCV GMCs, a single dose of either PCV10 or PCV13 elicited no response to the shared serotypes 6B, 14, and 23F, or to the non-PCV10 types 6A and 19A. After a single dose of either PCV10 or PCV13, more than half of participants had IgG concentrations of at least 0·35 μg/mL to serotypes 1, 4, 5, 7F, 14, and 19F in both groups, and to serotype 18C in the PCV13 group. Considering a 10% difference in the proportion of participants with IgG concentrations of at least 0·35 μg/mL as clinically significant, more participants had protective concentrations of IgG specific to serotype 19F in the PCV10 group than in the PCV13 group (risk difference 18·3% [11·4 to 25·2]), and more to serotype 18C in the PCV13 group than in the PCV10 group (risk difference −33·0% [–41·7 to −23·6]; appendix). Comparing the magnitude of the response (based on the ratio of GMCs for the ten shared serotypes), GMCs were higher in the PCV10 group for serotypes 1, 4, 5, 9V, and 19F, and higher in the PCV13 group for serotypes 7F and 18C (appendix).

At 9 months of age, pre-booster and 5 months post-primary series, most participants still had protective concentrations of antibody to most of the ten shared serotypes, ranging from 75·4% (69·4 to 80·8) to 100·0% [98·4 to 100·0] in the PCV10 group, and 68·9% (62·4 to 74·8) to 99·1% (96·9 to 99·9) in the PCV13 group. The proportion of participants with protective concentrations of serotype-specific antibody was higher in the PCV10 group than in the PCV13 group for serotype 6B (risk difference 18·6% [12·4 to 24·9]), and higher in the PCV13 group than in the PCV10 group for serotype 5 (risk difference −18·4% [–24·8 to −12·0]). GMCs were higher in the PCV10 group for serotypes 6B, 18C, 19F, and 23F, and higher in the PCV13 group for serotypes 1, 5 and 7F, 9V, and 14 (appendix).

Post-booster, the proportion of participants with IgG concentrations of at least 0·35 μg/mL was more than 97% for all ten shared serotypes in both groups (appendix). In terms of GMCs, the same pattern was seen post-booster dose as post-primary series for most serotypes, with GMCs higher in the PCV10 group than in the PCV13 group for serotype 19F, and higher in the PCV13 group than in the PCV10 group for serotypes 1, 5, 7F, 9V, 14, and 23F. By contrast with the post-primary series results, post-booster GMCs were higher in the PCV10 group than in the PCV13 group for serotype 18C, and higher in the PCV13 group than in the PCV10 group for serotype 6B, with no difference between groups for serotype 4 (appendix).

At 18 months of age, the proportion of participants with protective IgG concentrations was still greater than 95% for serotypes 14 and 19F (both groups) and serotype 6B (PCV10 group), and greater than 59% for all other shared serotypes, with no between-group differences at the 10% level (appendix). Differences in GMCs were only seen for serotypes 18C and 19F, which showed higher concentrations in the PCV10 group than in the PCV13 group (appendix).

For the non-PCV10 serotypes (3, 6A, and 19A), IgG concentrations of at least 0·35 μg/mL were seen in more than 94% of PCV13 recipients post-primary series (table 2), and more than 99% of PCV13 recipients post-booster (appendix). The GMC to serotype 3 was similar post-primary series (table 2) and post-booster (appendix) whereas GMCs for serotypes 6A and 19A increased substantially. PCV10 also elicited responses to serotypes 6A and 19A post-booster, with more than 90% of participants achieving IgG concentrations of at least 0·35 μg/mL (appendix). GMCs to all three non-PCV10 serotypes were higher in the PCV13 group than in the PCV10 group at all timepoints, with the exception of serotype 6A at 3 months of age (4 weeks post-one PCV dose) and serotype 19A at 18 months of age, for which there were no differences between the vaccine groups (appendix). The proportion of infants with serotype-specific IgG concentrations of at least 1·00 μg/mL were also compared post-primary series and post-booster (appendix). Post-primary series, more participants in the PCV13 group than in the PCV10 group had protective antibody concentrations for serotypes 1 and 5, and more participants in the PCV10 group than in the PCV13 group had protective concentrations for serotype 6B at the 10% level. Post-booster, more participants in the PCV13 group than in the PCV10 group had protective concentrations for serotype 5.

Differences in opsonophagocytic responses after the primary series of PCV10 or PCV13 vaccinations (table 3) broadly reflected those seen in the IgG concentrations. Geometric mean opsonisation indices were higher in the PCV10 group than in the PCV13 group for serotypes 6B and 19F, and higher in the PCV13 group than in the PCV10 group for all other serotypes except 14 (based on the ratio of geometric mean opsonisation indexes for the ten shared serotypes). The proportions of participants with an opsonisation index of 8 or more (table 3) also reflected the proportions of those with protective IgG concentrations for most serotypes, albeit with some exceptions: for serotype 1, more than 97% of infants in both groups had protective IgG concentrations (table 2), whereas the proportions achieving an opsonisation index of 8 or more were 66·1% (57·1–74·4) in the PCV10 group and 87·9% (80·8–93·1) in the PCV13 group (table 3). A similar pattern was seen for serotype 9V in the PCV10 group, with only 80·6% (72·6–87·2) achieving an opsonisation index of at least 8, compared with 96·2% (92·9–98·2) having a protective IgG concentration. Only serotypes 1 and 9V had differences between the PCV10 and PCV13 groups at the 10% level, with higher proportions of patients in the PCV13 group having opsonisation indices of 8 or more (table 3).

**Table 3:** Functional antibody responses post-primary series and post-booster

	**Post-primary series**						**Post-booster**					
	Participants with opsonisation index ≥8, %			Geometric mean opsonisation index			Participants with opsonisation index ≥8, %			Geometric mean opsonisation index		
	PCV10 (n=124)	PCV13 (n=124*)	Risk difference†	PCV10 (n=124)	PCV13 (n=124*)	Ratio‡	PCV10 (n=121)	PCV13 (n=120)	Risk difference†	PCV10 (n=121)	PCV13 (n=120)	Ratio‡
**Shared PCV serotypes**												
1	66·1 (57·1 to 74·4)	87·9 (80·8 to 93·1)	−21·8 (−31·6 to −11·4)	22 (17 to 28)	52 (40 to 67)	0·42 (0·30 to 0·61)	90·9 (84·3 to 95·4)	95·0 (89·4 to 98·1)	−4·1 (−11·1 to 2·7)	145 (106 to 198)	164 (127 to 211)	0·88 (0·59 to 1·32)
4	100·0 (97·1 to 100·0)	100·0 (97·1 to 100·0)	0·0 (−3·0 to 3·0)	922 (820 to 1036)	1320 (1188 to 1465)	0·70 (0·60 to 0·82)	99·2 (95·5 to 100·0)	100·0 (97·0 to 100·0)	−0·8 (−4·5 to 2·3)	1280 (1072 to 1529)	1771 (1560 to 2011)	0·72 (0·58 to 0·90)
5	97·6 (93·1 to 99·5)	98·4 (94·3 to 99·8)	−0·8 (−5·4 to 3·6)	351 (286 to 430)	476 (394 to 575)	0·74 (0·56 to 0·97)	98·3 (94·2 to 99·8)	100·0 (97·0 to 100·0)	−1·7 (−5·8 to 1·7)	768 (627 to 941)	929 (802 to 1076)	0·83 (0·64 to 1·06)
6B	71·8 (63·0 to 79·5)	60·5 (51·3 to 69·1)	11·3 (−0·5 to 22·6)	59 (40 to 86)	28 (20 to 40)	2·10 (1·26 to 3·50)	96·7 (91·8 to 99·1)	95·8 (90·5 to 98·6)	0·9 (−4·6 to 6·5)	299 (224 to 399)	826 (592 to 1153)	0·36 (0·23 to 0·56)
7F	96·8 (91·9 to 99·1)	98·4 (94·3 to 99·8)	−1·6 (−6·5 to 2·9)	250 (182 to 343)	570 (418 to 778)	0·44 (0·28 to 0·68)	100·0 (97·0 to 100·0)	100·0 (97·0 to 100·0)	0·0 (−3·1 to 3·1)	484 (369 to 636)	1231 (938 to 1615)	0·39 (0·27 to 0·58)
9V	80·6 (72·6 to 87·2)	99·2 (95·6 to 100·0)	−18·5 (−26·4 to −11·5)	73 (52 to 102)	267 (200 to 357)	0·27 (0·18 to 0·43)	94·2 (88·4 to 97·6)	99·2 (95·4 to 100·0)	−5·0 (−10·7 to −0·2)	308 (217 to 436)	742 (566 to 974)	0·41 (0·27 to 0·64)
14	89·5 (82·7 to 94·3)	93·5 (87·7 to 97·2)	−4·0 (−11·4 to 3·1)	132 (92 to 191)	220 (153 to 316)	0·60 (0·36 to 1·00)	96·7 (91·8 to 99·1)	96·7 (91·7 to 99·1)	0·0 (−5·3 to 5·3)	394 (293 to 531)	454 (328 to 628)	0·87 (0·56 to 1·34)
18C	88·7 (81·8 to 93·7)	96·8 (91·9 to 99·1)	−8·1 (−15·1 to −1·5)	124 (88 to 175)	242 (189 to 309)	0·51 (0·34 to 0·78)	99·2 (95·5 to 100·0)	100·0 (97·0 to 100·0)	−0·8 (−4·5 to 2·3)	732 (564 to 950)	561 (446 to 706)	1·31 (0·92 to 1·84)
19F	100·0 (97·1 to 100·0)	99·2 (95·6 to 100·0)	0·8 (−2·3 to 4·4)	1217 (1078 to 1375)	856 (728 to 1008)	1·42 (1·16 to 1·74)	100·0 (97·0 to 100·0)	98·3 (94·1 to 99·8)	1·7 (−1·6 to 5·9)	1579 (1380 to 1807)	1095 (877 to 1367)	1·44 (1·11 to 1·87)
23F	58·9 (49·7 to 67·6)	76·6 (68·2 to 83·7)	−17·7 (−28·7 to −6·1)	29 (21 to 41)	53 (38 to 75)	0·54 (0·33 to 0·88)	91·7 (85·3 to 96·0)	100·0 (97·0 to 100·0)	−8·3 (−14·5 to −3·4)	149 (109 to 202)	689 (534 to 890)	0·22 (0·14 to 0·32)
**Additional PCV13 serotypes**												
3	0·0 (0·0 to 2·9)	92·7 (86·6 to 96·6)	−92·7 (−96·1 to −86·0)	4 (4 to 4)	41 (34 to 50)	0·10 (0·08 to 0·12)	3·3 (0·9 to 8·2)	88·3 (81·2 to 93·5)	−85·0 (−90·0 to −76·5)	4 (4 to 5)	54 (43 to 68)	0·08 (0·06 to 0·10)
6A	31·5 (23·4–40·4)	97·6 (93·1 to 99·5)	−66·1 (−73·8 to −56·4)	18 (12 to 26)	1392 (1106 to 1752)	0·01 (0·01 to 0·02)	66·9 (57·8 to 75·2)	100·0 (97·0 to 100·0)	−33·1 (−41·8 to −24·7)	118 (74 to 189)	3847 (3311 to 4468)	0·03 (0·02 to 0·05)
19A	35·5 (27·1 to 44·6)	95·2 (89·8 to 98·2)	−59·7 (−68·0 to −49·4)	9 (7 to 11)	139 (106 to 181)	0·06 (0·04 to 0·09)	61·2 (51·9 to 69·9)	99·2 (95·4 to 100·0)	−38·0 (−46·9 to −29·0)	25 (18 to 34)	587 (461 to 748)	0·04 (0·03 to 0·06)

Data are point estimate (95% CI) for the proportion of participants with a serotype-specific opsonisation index of 8 or more, and geometric mean opsonisation indices at 4 weeks post-primary series and 4 weeks post-booster dose in participants given a 2 + 1 schedule of PCV10 or PCV13. PCV10=ten-valent pneumococcal conjugate vaccine. PCV13=13-valent pneumococcal conjugate vaccine.

* n=123 for serotype 3 (one sample not tested because of insufficient sera).

† Risk difference is PCV10 – PCV13.

‡ Ratio is PCV10 / PCV13.

There were fewer between-group differences post-booster than post-primary series (table 3). Geometric mean opsonisation indices were higher in the PCV10 group than in the PCV13 group for serotype 19F, and higher in the PCV13 group than in the PCV10 group for serotypes 4, 6B, 7F, 9V, and 23F based on the ratio of geometric mean opsonisation indices. More than 90% of participants in both groups achieved an opsonisation index of at least 8 for the ten shared serotypes, including serotype 1, with no differences between groups at the 10% level.

PCV13 was immunogenic to each of the non-PCV10 serotypes after the primary series, with more than 92% of participants achieving an opsonisation index of at least 8, and increased responses were seen following the booster dose for serotypes 6A and 19A (table 3). As with the IgG responses, PCV10 generated little to no functional immunity for serotypes 3, 6A, and 19A post-primary series, but substantial opsonophagocytic responses to serotypes 6A and 19A were seen after the booster dose of PCV10 (table 3).

Reactogenicity information was analysed at 2 months, 4 months, and 9·5 months of age in the 2 + 1 PCV10 group (group C) and the 2 + 1 PCV13 group (group E), and at 2 months and 4 months of age in the control group (group F; table 4). Diary cards were collected from more than 96% of participants vaccinated at each timepoint. The incidences of erythema at the PCV and the DTaP-IPV-Hib-HepB vaccination sites were both low. The incidence of erythema at the PCV site did not differ between the PCV10 and PCV13 groups at any timepoint (p=0·395 at 2 months, p=0·939 at 4 months, and p=0·346 at 9·5 months), and was similar to that at the DTaP-IPV-Hib-HepB site. Co-administration of DTaP-IPV-Hib-HepB with either PCV10 or PCV13 had no effect on the incidence of erythema at the DTaP-IPV-Hib-HepB site (p=0·590 at 2 months, p=0·100 at 4 months, and p>0·999 at 9·5 months; table 4).

**Table 4:** Reactogenicity

		**2 months**			**4 months**			**9·5 months**		
		N	Any	Severe*	N	Any	Severe*	N	Any	Severe*
**Erythema**										
At PCV10 site		244	23 (9%)	2 (1%)	235	26 (11%)	1 (<1%)	218	19 (9%)	1 (<1%)
At PCV13 site		237	17 (7%)	0	222	23 (10%)	1 (<1%)	211	12 (6%)	0
At DTaP-IPV-Hib-HepB site										
	PCV10 group	244	13 (5%)	2 (1%)	236	21 (9%)	1 (<1%)	222	13 (6%)	1 (<1%)
	PCV13 group	240	18 (8%)	1 (<1%)	225	29 (13%)	0	211	11 (5%)	0
	Control group	192	11 (6%)	0	188	15 (8%)	2 (1%)	NA	NA	NA
**Fever**										
PCV10 and DTaP-IPV-Hib-HepB		237	104 (44%)	10 (4%)	235	102 (43%)	11 (5%)	225	87 (39%)	16 (7%)
PCV13 and DTaP-IPV-Hib-HepB		236	98 (42%)	9 (4%)	227	100 (44%)	20 (9%)	219	89 (41%)	21 (10%)
DTaP-IPV-Hib-HepB alone		186	35 (19%)	3 (2%)	187	18 (10%)	4 (2%)	NA	NA	NA

Data are n (%) and show participants reporting erythema at the vaccination site(s) and participants reporting axillary fever after vaccination at various timepoints among participants for whom data were available (N). 1809 diary cards were collected, of which 20 were excluded because they contained no data on erythema or fever. Otherwise, all available data contributed to the analysis. The maximum reported values for erythema and fever across days 0–4 were used. PCV10=ten-valent pneumococcal conjugate vaccine. PCV13=13-valent pneumococcal conjugate vaccine. DTaP-IPV-Hib-HepB=hexavalent diphtheria, tetanus, pertussis, polio, *Haemophilus influenzae* type b, and hepatitis B vaccine. NA=Not applicable.

* Severe erythema was defined by a diameter of more than 30 mm, and severe fever was defined as a temperature of 38·5°C or higher.

The incidence of axillary fever (≥37·5°C) following PCV vaccination ranged from 39% to 44% (4–10% for severe fever [≥38·5°C]; table 4). Fever and severe fever did not differ in incidence between PCV10 recipients and PCV13 recipients at any timepoint (p=0·880 at 2 months, p=0·190 at 4 months, and p=0·643 at 9·5 months). In the PCV13 group, the proportion of fevers categorised as severe at 4 months and at 9·5 months was higher than that at 2 months (p=0·019). The incidence of fever after co-administration of PCV and DTaP-IPV-Hib-HepB was significantly higher than the incidence after DTaP-IPV-Hib-HepB vaccination alone (p<0·0001 at 2 months and at 4 months).

135 participants from groups A–F were hospitalised during the trial, in a total of 163 admissions (appendix). The most common reasons for hospitalisation were acute respiratory infection (70 [43%] of 163) and acute gastroenteritis (29 [18%]). 156 (94%) hospitalisations were unrelated to vaccination, and all resolved without sequelae. The reasons for hospitalisation (p=0·750) and the causality (in relation to vaccination; p=0·098) were similar across groups (appendix). No participants were withdrawn as a result of harms, and none died during the trial.

## Discussion

PCVs are now in use in national immunisation programmes in 142 countries. Increasingly, countries are adopting a 2 + 1 schedule, with a two-dose primary series followed by a booster dose at or after 9 months of age. In this paper we present the results of the first head-to-head study comparing the two currently available PCVs in a 2 + 1 schedule, measuring both serotype-specific IgG and functional antibody levels to all 13 serotypes in PCV13. The immunological advantage of one vaccine over the other varied by serotype and by timepoint. The overall pattern that emerges is that PCV10 generally fares better for the shared serotypes after a single dose. After the two-dose primary series, responses to PCV13 are stronger, but wane similarly to PCV10 by 9 months of age. PCV13 produces stronger booster responses, but this effect is lost by 18 months of age.

Responses after a single dose allow us to judge protection in the interval between doses. This knowledge is important because many children will not present on time for the second dose, and because 1 + 1 schedules are currently under consideration.^16^ After a single dose of either PCV10 or PCV13, there was no response to some serotypes (6B, 14, and 23F, and non-PCV10 types 6A and 19A). However, for most other serotypes, the majority of children responded beyond the protective concentration of 0·35 μg/mL, consistent with the observation that there is some incomplete protection afforded to infants by a single dose. The magnitude of the response was greater with PCV10 for half of the shared serotypes.

Both vaccines produced strong responses post-primary series, with more than 95% of children responding to most serotypes (the exceptions being 6B and 23F, consistent with previous findings^17^, ^18^), although the magnitude of the response was greater with PCV13 for eight of the shared serotypes. After the booster, almost all children had protective levels of antibody, but again the magnitude of the response was greater with PCV13 for seven of the shared serotypes. The concentration of 0·35 μg/mL was determined from a pooled analysis of data from efficacy trials, and was established as the basis for comparing new with existing PCVs post-primary series.^19^ The true protective concentration of antibody varies geographically, by serotype, and by disease type.^20^, ^21^, ^22^ Applying a more conservative concentration threshold of 1·00 μg/mL to our data, more than 80% of children responded to most serotypes post-primary series (the exceptions being serotypes 6B and 23F in both groups, and 5 in the PCV10 group), and more than 90% post-booster (the only exception being serotype 5 in the PCV10 group). At this threshold, PCV13 fared better for serotypes 1 (post-primary) and 5 (both post-primary and post-booster), and PCV10 for serotype 6B (post-primary).

In general, the opsonophagocytic assay titres reflected the ELISA titres, with similar proportions of infants protected by an IgG concentration of at least 0·35 μg/mL and infants with an opsonisation index of at least 8, but some important differences did emerge. With both vaccines, particularly PCV10, poor opsonophagocytic assay responses to serotype 1 were seen post-primary series, despite strong ELISA responses. This finding was reflected in the two European trials of investigational PCVs, in which 41% and 62% of participants in the PCV10 groups and 61% and 84% in the PCV13 groups had an opsonisation index of at least 8.^9^, ^10^ This disconnect between responses measured by opsonophagocytic assay and by ELISA is corrected after the booster dose, providing immunological evidence for the importance of a booster dose in protecting against disease. This is an important finding for Africa, where serotype 1 is a frequent cause of pneumococcal disease,^23^ and where most countries use a 3 + 0 schedule without a booster dose. Analysis of serotype 1 immunogenicity in the context of reduced-dose PCV10 schedules with or without a booster will be reported elsewhere as part of the evaluation of different PCV schedules (the other aim of this trial).

Both vaccines were strongly immunogenic against serotype 19F; however, responses were stronger after PCV10 vaccination at all timepoints and according to both ELISA and opsonophagocytic assay. By contrast, findings from the Dutch study^11^ showed that PCV13 produced stronger 19F booster responses by ELISA than did PCV10, although opsonophagocytic assay responses were similar. Serotype 19F has persisted in both carriage^24^ and disease^25^ in the USA, despite more than 15 years of vaccination, and has been the most common cause of vaccine failure in children.^26^ In the original PCV7 efficacy trial,^27^ effectiveness against invasive pneumococcal disease and ear disease for serotype 19F was lower than for other serotypes (along with serotype 6B), despite good circulating antibody levels. The sharp rise in serotype 19A disease after PCV7 introduction shows that the 19F component of PCV7 (and PCV13) provides no protection against 19A disease. By contrast, the 19F component of PCV10 appears to provide protection against 19A disease, although probably not carriage.^28^, ^29^

PCV13 elicited strong responses to the non-PCV10 serotypes, with more than 94% of children responding post-primary series and more than 99% post-booster. Interestingly, PCV13 produced only modest increases in IgG and opsonophagocytic assay responses for serotype 3 post-booster compared with post-primary series, and these responses were considerably lower than those for other serotypes, a finding consistent with previous immunogenicity data.^18^ The effectiveness of PCV13 against serotype 3 disease is in doubt.^30^, ^31^ Among PCV10 recipients, we found modest immunogenicity to serotypes 6A and 19A after the booster dose at 9 months, with more than 90% of children achieving an IgG concentration of at least 0·35 μg/mL, although the GMCs were significantly lower than those generated by PCV13. Opsonophagocytic assay responses were also lower but considerable. These results support findings from three experimental PCVs in the 1990s showing poor correlation between ELISA and opsonophagocytic assay results for cross-reactive serotypes,^32^ but are consistent with some degree of protection afforded by PCV10 against both 6A and 19A disease. As part of this trial, we are evaluating the effects of vaccination on pneumococcal carriage, which will elucidate the capacity for PCV10 to protect against carriage of serotypes 6A and 19A.

One of the limitations of this study was the use of immunological endpoints rather than disease outcomes. However, given that both PCV10 and PCV13 have been in routine use in many countries for several years with demonstrated effectiveness, a direct comparison of the two vaccines on this basis is appropriate, and is enhanced by the inclusion of functional opsonophagocytic assays in addition to the standard IgG antibody measurement by ELISA. Another limitation is the inclusion of assessment of responses to multiple serotypes at several timepoints, leading to the likelihood that some of the observed differences arose by chance. This is a problem faced by all studies of PCVs. To compensate for this, we defined a single conclusion for the primary outcome, requiring a difference (or non-inferiority) in the proportion of participants with an IgG concentration of at least 0·35 μg/mL to be observed for seven of the ten shared serotypes. Beyond the primary outcome, no formal adjustments for multiple comparisons were made. The inclusion of multiple outcomes in this study is also a strength. We have assessed the immunogenicity, with both ELISAs and opsonophagocytic assays, and reactogenicity of PCV10 and PCV13 in a 2 + 1 schedule, providing a comprehensive head-to-head comparison of these vaccines. For the reactogenicity assessments, a limitation of this study is the use of parent-held diary cards. However, the same potential issues of bias in self-reported symptoms apply to all study groups, and therefore would not affect the between-group comparisons. Furthermore, we reported a single measure for the occurrence of erythema and fever on days 0–4 post-vaccination to limit any effect of missing data; only 1% of diary cards were excluded from this analysis because of a lack of data.

In conclusion, PCV10 and PCV13 are highly immunogenic, consistent with their effectiveness, and show similar reactogenicity. The differences in immunogenicity described vary by serotype and timepoint. PCV13 tends to produce stronger responses post-primary series and post-booster, while PCV10 appears to produce stronger responses after a single dose. PCV10 produces reasonable responses to non-PCV10 types 6A and 19A, whereas PCV13 produces only modest responses to serotype 3. It has been argued that a higher antibody concentration is required to protect against mucosal disease than against invasive pneumococcal disease, but it is hard to assess whether or not the observed differences in immunogenicity would translate to differing degrees of protection afforded by the two vaccines. Further analysis of data from this trial will compare B-cell memory induced by PCV10 and PCV13 and will evaluate the effects of the two vaccines on the carriage of vaccine serotypes, vaccine-related serotypes, and other serotypes of pneumococcus, which might further elaborate the differences between the two vaccines.

All authors receive salary support from grants from the National Health and Medical Research Council of Australia or the Bill & Melinda Gates Foundation. Non-financial support (in the form of PCV10 vaccine doses) and funding for opsonophagocytic assays was provided by GlaxoSmithKline Biologicals SA. We declare no other competing interests.

## Data sharing

The study protocol and informed consent form have been published previously and are freely available. Data will be made publicly available in accordance with the rules set out by the Bill & Melinda Gates Foundation.
