# Supplementary material for: Immunogenicity and reactogenicity of ten-valent versus 13-valent pneumococcal conjugate vaccines among infants in Ho Chi Minh City, Vietnam: a randomised controlled trial
Source: Lancet Infect Dis. 2019 May;19(5):497–509. doi: 10.1016/S1473-3099(18)30734-5 (PMC6484092; doi:10.1016/S1473-3099(18)30734-5)
Supplement: Supplementary appendix [file mmc1.pdf]

THE LANCET
Infectious Diseases

Supplementary appendix

This appendix formed part of the original submission and has been peer reviewed. We post it as supplied by the authors.

Supplement to: Temple B, Toan NT, Dai VTT, et al. Immunogenicity and reactogenicity of ten-valent versus 13-valent pneumococcal conjugate vaccines among infants in Ho Chi Minh City, Vietnam: a randomised controlled trial. *Lancet Infect Dis* 2019; published online April 8. http://dx.doi.org/10.1016/S1473-3099(18)30734-5.

LIST OF APPENDIX TABLES

Table S1: Schedule of vaccines and samples for infants enrolled into the Vietnam Pneumococcal Project 2

Table S2: Post-primary series immunogenicity on the intention-to-treat population 3

Table S3: Comparison of responses to a single dose of PCV10 or PCV13 4

Table S4: Pre- and post-booster responses to a 2+1 schedule of PCV10 or PCV13 5

Table S5: Antibody levels at 18 months of age 6

Table S6: Percentage of participants with serotype-specific IgG ≥1.0μg/mL 7

Table S7: Hospitalisations 8

Table S1: Schedule of vaccines and samples for infants enrolled into the Vietnam Pneumococcal Project

Group	2m	3m	4m	5m	6m	7m	9m	9·5m†	10m	12m	18m	19m	24m
**A**<br>(3+1 PCV10)	Bld*<br>NP<br>PCV10<br>*Hexa*	PCV10<br>*Hexa*	PCV10<br>*Hexa*	Bld	NP		Bld<br>NP<br>PCV10<br>MV		Bld	NP	Bld*<br>NP<br>MR	*Hexa*	NP
**B**<br>(3+0 PCV10)	NP<br>PCV10<br>*Hexa*	PCV10<br>*Hexa*	PCV10<br>*Hexa*	Bld	Bld<br>NP		Bld*<br>NP<br>MV		Bld	NP	Bld*<br>NP<br>MR	*Hexa*	NP
**C**<br>(2+1 PCV10)	NP<br>PCV10<br>*Hexa*		PCV10<br>*Hexa*	Bld	Bld*<br>NP		Bld<br>NP<br>MV	PCV10<br>*Hexa*	Bld	NP	Bld*<br>NP<br>MR	*Hexa*	NP
**D**<br>(2-dose PCV10)	NP<br>PCV10<br>*Hexa*	Bld	*Hexa*		Bld<br>NP<br>PCV10<br>*Hexa*	Bld	Bld*<br>NP<br>MV			NP	Bld*<br>NP<br>MR	*Hexa*	NP
**E**<br>(2+1 PCV13)	NP<br>PCV13<br>*Hexa*	Bld*	PCV13<br>*Hexa*	Bld	NP		Bld<br>NP<br>MV	PCV13<br>*Hexa*	Bld	NP	Bld*<br>NP<br>MR	*Hexa*	NP
**F**<br>(controls)	NP<br>*Hexa*	*Hexa*	*Hexa*		NP		NP<br>MV			NP	Bld<br>NP<br>PCV10<br>*Hexa*	Bld<br>MR	NP<br>Bld<br>PCV10

Bld = blood sample. NP = nasopharyngeal swab sample. PCV10 = ten-valent pneumococcal conjugate vaccine. PCV13 = 13-valent pneumococcal conjugate vaccine. *Hexa* = hexavalent diphtheria, tetanus, pertussis, polio, Haemophilus influenzae type b, and hepatitis B vaccine (DTaP-IPV-Hib-HepB). MV = measles vaccine. MR = measles-rubella vaccine.

* Each participant provides only one of these blood samples (participants allocated to groups A-E from the last 300 recruited provide this sample at 18 months of age; the remainder provide it at the other time point).

† The Vietnam Ministry of Health does not permit co-administration of measles and DTaP-IPV-Hib-HepB; therefore PCV and DTaP-IPV-Hib-HepB were administered at 9·5 months in participants from groups C and E.

Table S2: Post-primary series immunogenicity on the intention-to-treat population

Immunogenicity data at 4 weeks after two doses of PCV10 (at 2 months and 4 months of age, group C), two doses of PCV13 (at 2 months and 4 months of age, group E), or three doses of PCV10 (at 2 months, 3 months, and 4 months of age, group A and B). GMC=geometric mean concentration. PCV10=ten-valent pneumococcal conjugate vaccine. PCV13=13-valent pneumococcal conjugate vaccine.

	**Participants with IgG concentration ≥0·35µg/mL, % (95% CI)**			**Risk difference, %**		**GMC, µg/mL (95% CI)**			**GMC ratio (95% CI)**	
	Two-dose PCV10 (n=240)	Three-dose PCV10 (n=289)	Two-dose PCV13 (n=236)	Two-dose PCV10 to PCV13 (95% CI)	Three-dose PCV10 to PCV13 (90% CI)	Two-dose PCV10 (n=240)	Three-dose PCV10 (n=289)	Two-dose PCV13 (n=236)	Two-dose PCV10/PCV13	Three-dose PCV10/PCV13
Shared PCV serotypes										
1	97.9	98.3	100	-2.1	-1.7	2.22	2.78	4.86	0.46*	0.57*
	(95.2, 99.3)	(96.0, 99.4)	(98.4, 100)	(-4.8, -0.1)	(-3.5, -0.3)	(1.98, 2.48)	(2.51, 3.08)	(4.38, 5.39)	(0.39, 0.53)	(0.49, 0.66)
4	98.8	99.0	100	-1.3	-1	3.21	3.85	4.79	0.67*	0.8*
	(96.4, 99.7)	(97.0, 99.8)	(98.4, 100)	(-3.6, 0.6)	(-2.6, 0.3)	(2.88, 3.58)	(3.45, 4.30)	(4.39, 5.23)	(0.58, 0.77)	(0.69, 0.93)
5	95.8	98.6	99.2	-3.3	-0.5	1.17	1.82	2.2	0.53*	0.83*
	(92.5, 98.0)	(96.5, 99.6)	(97.0, 99.9)	(-6.7, -0.4)	(-2.3, 1.3)	(1.07, 1.27)	(1.67, 1.98)	(2.01, 2.41)	(0.47, 0.60)	(0.73, 0.94)
6B	77.1	84.4	61.0	16.1	23.4‡	0.8	1.08	0.49	1.65†	2.22†
	(71.2, 82.2)	(79.7, 88.4)	(54.5, 67.3)	(7.8, 24.1)	(17.1, 29.6)	(0.70, 0.92)	(0.95, 1.23)	(0.43, 0.55)	(1.37, 2.00)	(1.84, 2.66)
7F	98.8	99.3	100	-1.3	-0.7	2.07	3.03	3.31	0.63*	0.92
	(96.4, 99.7)	(97.5, 99.9)	(98.4, 100)	(-3.6, 0.6)	(-2.1, 0.5)	(1.89, 2.26)	(2.78, 3.30)	(3.03, 3.60)	(0.55, 0.71)	(0.81, 1.04)
9V	96.3	99.3	97.9	-1.6	1.4	1.63	2.47	3.24	0.50*	0.76*
	(93.0, 98.3)	(97.5, 99.9)	(95.1, 99.3)	(-5.1, 1.6)	(-0.3, 3.6)	(1.47, 1.81)	(2.26, 2.70)	(2.91, 3.62)	(0.43, 0.58)	(0.66, 0.88)
14	98.3	100	98.3	0	1.7	5.92	9.72	7.83	0.76*	1.24†
	(95.8, 99.5)	(98.7, 100)	(95.7, 99.5)	(-2.7, 2.8)	(0.4, 3.7)	(5.17, 6.78)	(8.76, 10.79)	(6.68, 9.18)	(0.61, 0.93)	(1.03, 1.49)
18C	96.7	98.6	98.7	-2.1	-0.1	1.87	3.86	3.12	0.60*	1.24†
	(93.5, 98.6)	(96.5, 99.6)	(96.3, 99.7)	(-5.3, 0.8)	(-1.9, 1.9)	(1.65, 2.12)	(3.47, 4.29)	(2.82, 3.45)	(0.51, 0.70)	(1.07, 1.44)
19F	99.2	99.7	99.2	0	0.5	9.56	8.22	7.6	1.26†	1.08
	(97.0, 99.9)	(98.1, 100)	(97.0, 99.9)	(-2.2, 2.3)	(-0.8, 2.2)	(8.40, 10.88)	(7.42, 9.12)	(6.72, 8.59)	(1.05, 1.50)	(0.92, 1.27)
23F	77.9	90.3	89.4	-11.5	0.9	0.89	1.32	1.14	0.78*	1.16
	(72.1, 83.0)	(86.3, 93.5)	(84.8, 93.0)	(-18.1, -4.9)	(-3.4, 5.4)	(0.78, 1.02)	(1.18, 1.48)	(1.01, 1.29)	(0.65, 0.94)	(0.98, 1.37)
Additional PCV13 serotypes										
3	5.8	6.9	97.9	-92	-91	0.1	0.11	1.54	0.07	0.07
	(3.2, 9.6)	(4.3, 10.5)	(95.1, 99.3)	(-94.7, -87.4)	(-93.3, -87.4)	(0.09, 0.11)	(0.10, 0.12)	(1.41, 1.68)	(0.06, 0.08)	(0.06, 0.08)
6A	40.8	50.5	94.9	-54.1	-44.4	0.31	0.37	1.94	0.16	0.19
	(34.6, 47.3)	(44.6, 56.4)	(91.3, 97.3)	(-60.5, -46.8)	(-49.6, -38.8)	(0.28, 0.35)	(0.34, 0.41)	(1.70, 2.22)	(0.14, 0.19)	(0.16, 0.22)
19A	70	67.8	98.3	-28.3	-30.5	0.55	0.56	3.8	0.14	0.15
	(63.8, 75.7)	(62.1, 73.2)	(95.7, 99.5)	(-34.5, -22.3)	(-35.2, -25.7)	(0.49, 0.61)	(0.50, 0.62)	(3.33, 4.33)	(0.12, 0.17)	(0.13, 0.17)

* indicates a GMC ratio with a 95% CI excluding 1.00, PCV13 higher
† indicates a GMC ratio with a 95% CI excluding 1.00, PCV10 higher
‡ indicates a risk difference with upper bound of the 90% CI ≥10%

Table S3: Comparison of responses to a single dose of PCV10 or PCV13

Immunogenicity data before and at 4 weeks after a single dose of PCV at 2 months of age. GMC=geometric mean concentration. PCV10=ten-valent pneumococcal conjugate vaccine. PCV13=13-valent pneumococcal conjugate vaccine.

	**Participants with IgG concnetration ≥0·35μg/mL, % (95% CI)**			**Risk difference (95% CI)**	**GMC, μg/mL (95% CI)**			**GMC ratio (95% CI)**
	Pre-PCV (n=100)	Post-PCV10 (n=197)	Post-PCV13 (n=193)	Post-PCV (PCV10-PCV13)	Pre-PCV (n=100)	Post-PCV10 (n=197)	Post-PCV13 (n=193)	Post-PCV (PCV10/PCV13)
Shared serotypes								
1	14·0	88·3	73·1	15·3	0·12	1·05	0·64	1·64†
	(7·9, 22·4)	(83·0, 92·5)	(66·2, 79·2)	(7·5, 22·9)	(0·10, 0·15)	(0·91, 1·20)	(0·56, 0·73)	(1·35, 1·99)
4	8·0	88·8	82·9	5·9	0·09	1·12	0·88	1·28†
	(3·5, 15·2)	(83·6, 92·9)	(76·8, 87·9)	(-1·0, 12·9)	(0·07, 0·10)	(0·98, 1·29)	(0·77, 1·00)	(1·06, 1·55)
5	10·0	79·7	64·2	15·4	0·11	0·85	0·46	1·83†
	(4·9, 17·6)	(73·4, 85·1)	(57·0, 71·0)	(6·5, 24·0)	(0·10, 0·13)	(0·74, 0·97)	(0·40, 0·53)	(1·51, 2·23)
6B	22·0	15·7	14·0	1·7	0·21	0·18	0·17	1·02
	(14·3, 31·4)	(10·9, 21·6)	(9·4, 19·7)	(-5·4, 8·9)	(0·18, 0·24)	(0·16, 0·20)	(0·15, 0·19)	(0·87, 1·20)
7F	10·0	70·6	80·8	-10·3	0·11	0·57	0·81	0·71*
	(4·9, 17·6)	(63·7, 76·8)	(74·6, 86·1)	(-18·6, -1·7)	(0·09, 0·13)	(0·50, 0·66)	(0·70, 0·94)	(0·58, 0·86)
9V	17·0	49·2	35·2	14·0	0·18	0·35	0·28	1·25†
	(10·2, 25·8)	(42·1, 56·4)	(28·5, 42·4)	(4·2, 23·4)	(0·15, 0·20)	(0·31, 0·39)	(0·25, 0·31)	(1·07, 1·46)
14	68·0	77·2	72·5	4·6	0·64	0·69	0·65	1·06
	(57·9, 77·0)	(70·7, 82·8)	(65·7, 78·7)	(-4·0, 13·2)	(0·49, 0·84)	(0·60, 0·78)	(0·55, 0·76)	(0·86, 1·30)
18C	26·0	44·2	77·2	-33·0§	0·24	0·34	0·62	0·54*
	(17·7, 35·7)	(37·1, 51·4)	(70·6, 82·9)	(-41·7, -23·6)	(0·21, 0·28)	(0·30, 0·38)	(0·55, 0·70)	(0·46, 0·65)
19F	66·0	94·4	76·2	18·3‡	0·45	1·09	0·58	1·87†
	(55·8, 75·2)	(90·2, 97·2)	(69·5, 82·0)	(11·4, 25·2)	(0·39, 0·53)	(0·97, 1·21)	(0·53, 0·64)	(1·62, 2·16)
23F	23·0	13·2	15·0	-1·8	0·19	0·16	0·15	1·04
	(15·2, 32·5)	(8·8, 18·7)	(10·3, 20·9)	(-8·8, 5·1)	(0·17, 0·23)	(0·14, 0·18)	(0·13, 0·17)	(0·88, 1·23)
Additional PCV13-types								
3	5·0	2·0	88·1	-86·1	0·07	0·06	0·80	0·07
	(1·6, 11·3)	(0·6, 5·1)	(82·7, 92·3)	(-90·1, -79·9)	(0·06, 0·09)	(0·05, 0·07)	(0·72, 0·89)	(0·06, 0·09)
6A	44·0	27·4	31·1	-3·7	0·32	0·25	0·25	0·99
	(34·1, 54·3)	(21·3, 34·2)	(24·6, 38·1)	(-12·6, 5·3)	(0·28, 0·37)	(0·23, 0·28)	(0·23, 0·28)	(0·87, 1·14)
19A	61·0	46·2	60·1	-13·9	0·41	0·33	0·43	0·79
	(50·7, 70·6)	(39·1, 53·4)	(52·8, 67·1)	(-23·4, -4·0)	(0·36, 0·47)	(0·30, 0·37)	(0·38, 0·47)	(0·68, 0·91)

* indicates a GMC ratio with a 95% CI excluding 1.00, PCV13 higher
† indicates a GMC ratio with a 95% CI excluding 1.00, PCV10 higher
‡ indicates a risk difference with 95% CI entirely below -10% (PCV13 better)
§ indicates a risk difference with 95% CI entirely above 10% (PCV10 better)

Table S4: Pre- and post-booster responses to a 2+1 schedule of PCV10 or PCV13

Immunogenicity data before and at 4 weeks after a booster dose of PCV at 9.5 months of age. GMC=geometric mean concentration. PCV10=ten-valent pneumococcal conjugate vaccine. PCV13=13-valent pneumococcal conjugate vaccine.

a) Participants with serotype-specific IgG ≥0·35µg/mL before and at 4 weeks after a booster dose of PCV at 9·5 months of age, % (95% CI)

	PCV10		PCV13		Pre-booster risk difference (95% CI) (PCV10-PCV13)	Post-booster risk difference (95% CI) (PCV10-PCV13)
	Pre-booster (n=236)	Post-booster (n=226)	Pre-booster (n=228)	Post-booster (n=221)		
Shared serotypes						
1	82·6 (77·2, 87·2)	100 (98·4, 100)	96·5 (93·2, 98·5)	100 (98·3, 100)	-13·9 (-19·5, -8·5)	0 (-1·7, 1·7)
4	91·1 (86·7, 94·4)	98·7 (96·2, 99·7)	96·5 (93·2, 98·5)	100 (98·3, 100)	-5·4 (-10·0, -1·0)	-1·3 (-3·8, 0·6)
5	75·4 (69·4, 80·8)	97·8 (94·9, 99·3)	93·9 (89·9, 96·6)	99·5 (97·5, 100)	-18·4 (-24·8, -12·0)‡	-1·8 (-4·6, 0·7)
6B	94·9 (91·3, 97·3)	100 (98·4, 100)	76·3 (70·3, 81·7)	98·2 (95·4, 99·5)	18·6 (12·4, 24·9)§	1·8 (-0·2, 4·6)
7F	90·3 (85·7, 93·7)	99·6 (97·6, 100)	94·7 (91·0, 97·3)	100 (98·3, 100)	-4·5 (-9·5, 0·4)	-0·4 (-2·5, 1·3)
9V	86·0 (80·9, 90·2)	100 (98·4, 100)	94·3 (90·4, 96·9)	99·5 (97·5, 100)	-8·3 (-13·8, -2·9)	0·5 (-1·3, 2·5)
14	97·5 (94·5, 99·1)	99·6 (97·6, 100)	97·4 (94·4, 99·0)	100 (98·3, 100)	0·1 (-3·1, 3·4)	-0·4 (-2·5, 1·3)
18C	84·7 (79·5, 89·1)	100 (98·4, 100)	88·6 (83·7, 92·4)	99·5 (97·5, 100)	-3·9 (-10·1, 2·4)	0·5 (-1·3, 2·5)
19F	100 (98·4, 100)	100 (98·4, 100)	99·1 (96·9, 99·9)	100 (98·3, 100)	0·9 (-0·8, 3·1)	0 (-1·7, 1·7)
23F	83·1 (77·6, 87·6)	98·7 (96·2, 99·7)	68·9 (62·4, 74·8)	99·5 (97·5, 100)	14·2 (6·4, 21·8)	-0·9 (-3·4, 1·4)
Additional PCV13-types						
3	13·1 (9·1, 18·1)	31·0 (25·0, 37·4)	72·8 (66·5, 78·5)	99·1 (96·8, 99·9)	-59·7 (-66·2, -51·8)	-68·1 (-73·8, -61·4)
6A	69·9 (63·6, 75·7)	91·6 (87·2, 94·9)	94·7 (91·0, 97·3)	99·5 (97·5, 100)	-24·8 (-31·3, -18·2)	-8·0 (-12·3, -4·3)
19A	78·8 (73·0, 83·8)	95·6 (92·0, 97·9)	96·5 (93·2, 98·5)	100 (98·3, 100)	-17·7 (-23·6, -11·9)	-4·4 (-8·0, -1·8)

b) GMCs before and at 4 weeks after a booster dose of PCV at 9·5 months of age, µg/mL (95% CI)

	PCV10		PCV13		Pre-booster GMC ratio (95% CI) (PCV10/PCV13)	Post-booster GMC ratio (95% CI) (PCV10/PCV13)
	Pre-booster (n=236)	Post-booster (n=226)	Pre-booster (n=228)	Post-booster (n=221)		
Shared serotypes						
1	0·71 (0·64, 0·79)	4·40 (3·91, 4·97)	1·40 (1·28, 1·53)	7·62 (6·86, 8·45)	0·51 (0·44, 0·58)*	0·58 (0·49, 0·68)*
4	1·09 (0·98, 1·22)	4·75 (4·20, 5·37)	1·14 (1·04, 1·24)	5·32 (4·82, 5·87)	0·96 (0·83, 1·11)	0·89 (0·76, 1·04)
5	0·54 (0·49, 0·59)	1·31 (1·20, 1·43)	0·85 (0·78, 0·92)	3·31 (3·00, 3·66)	0·63 (0·56, 0·72)*	0·40 (0·35, 0·45)*
6B	1·63 (1·44, 1·83)	6·17 (5·50, 6·92)	0·63 (0·56, 0·70)	9·51 (8·16, 11·09)	2·60 (2·21, 3·05)†	0·65 (0·54, 0·78)*
7F	0·83 (0·76, 0·91)	2·65 (2·41, 2·91)	1·07 (0·98, 1·17)	4·76 (4·33, 5·24)	0·78 (0·68, 0·88)*	0·56 (0·49, 0·64)*
9V	0·75 (0·68, 0·84)	3·34 (3·02, 3·69)	0·91 (0·83, 1·00)	5·23 (4·75, 5·77)	0·82 (0·72, 0·95)*	0·64 (0·55, 0·73)*
14	3·41 (2·96, 3·94)	11·76 (10·45, 13·24)	4·43 (3·89, 5·05)	15·37 (13·73, 17·21)	0·77 (0·63, 0·94)*	0·77 (0·65, 0·90)*
18C	0·81 (0·72, 0·90)	5·16 (4·68, 5·70)	0·67 (0·62, 0·73)	4·31 (3·89, 4·79)	1·19 (1·04, 1·37)†	1·20 (1·04, 1·38)†
19F	3·94 (3·59, 4·31)	16·16 (14·45, 18·08)	2·16 (1·97, 2·37)	11·68 (10·48, 13·02)	1·82 (1·60, 2·08)†	1·38 (1·18, 1·62)†
23F	0·76 (0·68, 0·86)	3·55 (3·15, 3·99)	0·51 (0·46, 0·57)	6·12 (5·40, 6·94)	1·49 (1·27, 1·75)†	0·58 (0·49, 0·69)*
Additional PCV13-types						
3	0·15 (0·13, 0·16)	0·25 (0·23, 0·29)	0·48 (0·45, 0·51)	1·82 (1·65, 2·01)	0·31 (0·27, 0·35)	0·14 (0·12, 0·16)
6A	0·57 (0·51, 0·65)	1·44 (1·25, 1·66)	1·18 (1·06, 1·31)	9·13 (7·99, 10·43)	0·49 (0·42, 0·57)	0·16 (0·13, 0·19)
19A	0·66 (0·60, 0·73)	1·76 (1·55, 2·00)	1·24 (1·11, 1·39)	9·18 (8·16, 10·33)	0·53 (0·46, 0·61)	0·19 (0·16, 0·23)

* indicates a GMC ratio with a 95% CI excluding 1.00, PCV13 higher
† indicates a GMC ratio with a 95% CI excluding 1.00, PCV10 higher
‡ indicates a risk difference with 95% CI entirely below -10% (PCV13 better)
§ indicates a risk difference with 95% CI entirely above 10% (PCV10 better)

Table S5: Antibody levels at 18 months of age

Immunogenicity data in a subset of participants at 18 months of age, following a 2+1 schedule of PCV10 or PCV13 at 2, 4, and 9·5 months of age. GMC=geometric mean concentration. PCV10=ten-valent pneumococcal conjugate vaccine. PCV13=13-valent pneumococcal conjugate vaccine.

	**Participants with IgG concentration ≥0·35µg/mL, % (95% CI)**		**Risk difference (95% CI)** (PCV10-PCV13)	**GMC, µg/mL (95% CI)**		**GMC ratio (95% CI)** (PCV10/PCV13)
	PCV10 (n=47)	PCV13 (n=46)		PCV10 (n=47)	PCV13 (n=46)	
Shared serotypes						
1	76·6 (62·0, 87·7)	87·0 (73·7, 95·1)	-10·4 (-25·8, 5·6)	0·68 (0·53, 0·87)	0·77 (0·61, 0·96)	0·88 (0·63, 1·23)
4	72·3 (57·4, 84·4)	63·0 (47·5, 76·8)	9·3 (-9·5, 27·3)	0·56 (0·45, 0·71)	0·43 (0·34, 0·54)	1·31 (0·96, 1·79)
5	80·9 (66·7, 90·9)	78·3 (63·6, 89·1)	2·6 (-13·8, 19·0)	0·61 (0·49, 0·74)	0·56 (0·44, 0·70)	1·09 (0·80, 1·47)
6B	95·7 (85·5, 99·5)	87·0 (73·7, 95·1)	8·8 (-3·4, 21·8)	1·15 (0·87, 1·54)	1·32 (0·93, 1·86)	0·88 (0·56, 1·36)
7F	59·6 (44·3, 73·6)	73·9 (58·9, 85·7)	-14·3 (-32·0, 4·7)	0·46 (0·35, 0·59)	0·53 (0·43, 0·66)	0·86 (0·62, 1·20)
9V	83·0 (69·2, 92·4)	69·6 (54·2, 82·3)	13·4 (-3·9, 29·9)	0·55 (0·45, 0·67)	0·45 (0·36, 0·58)	1·21 (0·89, 1·64)
14	97·9 (88·7, 99·9)	97·8 (88·5, 99·9)	0·0 (-9·1, 9·4)	1·94 (1·49, 2·52)	1·67 (1·27, 2·20)	1·16 (0·80, 1·69)
18C	74·5 (59·7, 86·1)	60·9 (45·4, 74·9)	13·6 (-5·3, 31·3)	0·67 (0·53, 0·86)	0·36 (0·28, 0·46)	1·85 (1·32, 2·61)†
19F	100 (92·5, 100)	95·7 (85·2, 99·5)	4·3 (-3·8, 14·5)	3·36 (2·56, 4·40)	1·73 (1·31, 2·28)	1·94 (1·32, 2·86)†
23F	80·9 (66·7, 90·9)	78·3 (63·6, 89·1)	2·6 (-13·8, 19·0)	0·77 (0·59, 1·01)	0·95 (0·65, 1·38)	0·81 (0·51, 1·29)
Additional PCV13-types						
3	17·0 (7·6, 30·8)	39·1 (25·1, 54·6)	-22·1 (-38·7, -3·8)	0·14 (0·11, 0·18)	0·29 (0·22, 0·38)	0·47 (0·33, 0·67)
6A	74·5 (59·7, 86·1)	84·8 (71·1, 93·7)	-10·3 (-26·2, 6·3)	0·59 (0·46, 0·76)	1·12 (0·75, 1·68)	0·53 (0·33, 0·84)
19A	83·0 (69·2, 92·4)	93·5 (82·1, 98·6)	-10·5 (-24·3, 3·2)	0·86 (0·66, 1·13)	1·26 (0·94, 1·68)	0·69 (0·46, 1·01)

† indicates a GMC ratio with a 95% CI excluding 1.00, PCV10 higher

Table S6: Percentage of participants with serotype-specific IgG ≥1.0µg/mL

Post-hoc analysis of immunogenicity data at 4 weeks post-primary series and 4 weeks post-booster, in participants that received a 2+1 schedule of PCV10 or PCV13 at 2, 4, and 9·5 months of age. PCV10=ten-valent pneumococcal conjugate vaccine. PCV13=13-valent pneumococcal conjugate vaccine.

	**Post-primary series**			**Post-booster**		
	PCV10 (n=237)	PCV13 (n=232)	Risk difference (95% CI) (PCV10-PCV13)	PCV10 (n=226)	PCV13 (n=221)	Risk difference (95% CI) (PCV10-PCV13)
Shared serotypes						
1	81.4 (75.9, 86.2)	97 (93.9, 98.8)	-15.5 (-21.2, -10.1)§	92.5 (88.2, 95.6)	98.6 (96.1, 99.7)	-6.2 (-10.5, -2.4)
4	90.3 (85.8, 93.7)	98.7 (96.3, 99.7)	-8.4 (-12.9, -4.4)	95.1 (91.5, 97.5)	99.1 (96.8, 99.9)	-4 (-7.7, -0.8)
5	64.1 (57.7, 70.2)	84.9 (79.6, 89.3)	-20.8 (-28.2, -13.0)§	65.5 (58.9, 71.7)	95.9 (92.4, 98.1)	-30.4 (-37.1, -23.6)§
6B	42.2 (35.8, 48.8)	20.7 (15.7, 26.5)	21.5 (13.2, 29.4)‡	97.8 (94.9, 99.3)	94.6 (90.7, 97.2)	3.2 (-0.5, 7.2)
7F	86.9 (82.0, 90.9)	94.8 (91.1, 97.3)	-7.9 (-13.3, -2.7)	92.5 (88.2, 95.6)	99.1 (96.8, 99.9)	-6.6 (-10.9, -3.0)
9V	76.8 (70.9, 82.0)	91.8 (87.5, 95.0)	-15 (-21.5, -8.5)	96.5 (93.1, 98.5)	98.2 (95.4, 99.5)	-1.7 (-5.2, 1.5)
14	93.2 (89.3, 96.1)	93.5 (89.6, 96.3)	-0.3 (-5.0, 4.4)	99.1 (96.8, 99.9)	98.6 (96.1, 99.7)	0.5 (-2.0, 3.1)
18C	77.2 (71.3, 82.4)	93.1 (89.0, 96.0)	-15.9 (-22.2, -9.6)	99.6 (97.6, 100.0)	95.9 (92.4, 98.1)	3.6 (0.8, 7.1)
19F	95.4 (91.8, 97.7)	96.6 (93.3, 98.5)	-1.2 (-5.1, 2.6)	100 (98.4, 100.0)	99.1 (96.8, 99.9)	0.9 (-0.9, 3.2)
23F	50.2 (43.7, 56.7)	53.4 (46.8, 60.0)	-3.2 (-12.2, 5.8)	91.6 (87.2, 94.9)	96.4 (93.0, 98.4)	-4.8 (-9.5, -0.3)
Additional PCV13-types						
3	2.1 (0.7, 4.9)	77.6 (71.7, 82.8)	-75.5 (-80.5, -69.1)	9.7 (6.2, 14.4)	79.6 (73.7, 84.7)	-69.9 (-75.7, -62.5)
6A	10.5 (6.9, 15.2)	75 (68.9, 80.4)	-64.5 (-70.6, -57.0)	60.6 (53.9, 67.0)	97.7 (94.8, 99.3)	-37.1 (-43.7, -30.3)
19A	21.5 (16.5, 27.3)	89.2 (84.5, 92.9)	-67.7 (-73.5, -60.4)	73.5 (67.2, 79.1)	99.1 (96.8, 99.9)	-25.6 (-31.8, -19.8)

‡ indicates a risk difference with 95% CI entirely below -10% (PCV13 better)
§ indicates a risk difference with 95% CI entirely above 10% (PCV10 better)

Table S7: Hospitalisations

Number (%) of hospitalisations by study group, and breakdown of reason for hospitalisation and causality (in relation to vaccination), n (%) within each study group

	Group A	Group B	Group C	Group D	Group E	Group F	Total
All hospitalisations, n (%)	21 (13%)	22 (13%)	39 (24%)	29 (18%)	28 (17%)	24 (15%)	163
Reason for hospitalisation, n (%)							
Acute respiratory infection	12 (57%)	7 (32%)	16 (41%)	13 (45%)	13 (46.5%)	9 (38%)	70 (43%)
Acute gastroenteritis	3 (14%)	6 (27%)	7 (18%)	5 (17%)	6 (21.5%)	2 (8%)	29 (18%)
Other	6 (29%)	9 (41%)	16 (41%)	11 (38%)	9 (32%)	13 (54%)	64 (39%)
Causality, n (%)							
Unrelated to vaccination	20 (95%)	21 (95%)	38 (97%)	27 (93%)	26 (93%)	24 (100%)	156 (96%)
Unlikely related to vaccination	0 (0%)	0 (0%)	0 (0%)	0 (0%)	2 (7%)	0 (0%)	2 (1%)
Possibly related to vaccination	1 (5%)	0 (0%)	0 (0%)	2 (7%)	0 (0%)	0 (0%)	3 (2%)
Probably related to vaccination	0 (0%)	1 (5%)	0 (0%)	0 (0%)	0 (0%)	0 (0%)	1 (<1%)
Related to vaccination	0 (0%)	0 (0%)	1 (3%)	0 (0%)	0 (0%)	0 (0%)	1 (<1%)
